# Association between impulsivity and cognitive capacity decrease is mediated by smartphone addiction, academic procrastination, bedtime procrastination, sleep insufficiency and daytime fatigue among medical students: a path analysis

**DOI:** 10.1186/s12909-023-04522-8

**Published:** 2023-07-27

**Authors:** Csaba Hamvai, Hedvig Kiss, Henrietta Vörös, Kevin M. Fitzpatrick, András Vargha, Bettina F. Pikó

**Affiliations:** 1 Department of Behavioral Sciences, University of Szeged, Mars tér 20, 6722 Szeged, Hungary; 2 Department of Sociology & Criminology, University of Arkansas, Fayetteville, AR USA; 3 Institute of Psychology, Károli Gáspár Reformed Church University, Budapest, Hungary; 4 Institute of Psychology, Eötvös Loránad University, Budapest, Hungary

**Keywords:** Impulsivity, Smartphone addiction, Academic, Bedtime, Procrastination, Sleep outcomes, Medical student

## Abstract

**Background:**

Medical students are at high risk for sleep disturbance. One possible cause of their sleeping problem is impulsivity. We aim to investigate the possible mediators between medical students’ impulsivity and sleep outcomes. Thus, we developed and investigated a model where the predictors were attentional, non-planning, and motor impulsivity subtraits. In the final model, subjective cognitive capacity decrease was the outcome variable. In light of previous findings, academic procrastination, smartphone addiction, and bedtime procrastination were considered important mediators as well as two variables of poor sleep, sleeping insufficiency, and daytime fatigue.

**Methods:**

Medical students (*N* = 211; age_M_ = 22.15 years; age_SD_ = 3.47 years; 71.6% women) were recruited to complete an online survey comprised of demographics (age, gender), self-administered scales (Abbreviated Impulsiveness Scale, Bedtime Procrastination Scale, Abbreviated Impulsiveness Scale, Academic Procrastination Scale-Short Form) and questions on tiredness, daily fatigue and subjective cognitive capacity decrease. Correlation and path analyses were implemented to examine hypothesized relationships between the variables.

**Results:**

Both attentional impulsivity (β = 0.33,* p* < .001) and non-planning impulsivity (β = -0.19, *p* < .01) had a direct relationship with cognitive capacity decrease. Attentional impulsivity was also associated with decreased cognitive capacity with a serial mediation effect via smartphone addiction, academic procrastination, bedtime procrastination, sleep insufficiency and fatigue (estimate = 0.017, *p* < .01). The indirect link between non-planning impulsivity and cognitive capacity decrease was mediated by academic procrastination, bedtime procrastination, sleep insufficiency and fatigue (estimate = 0.011, *p* < .01).

**Conclusions:**

Inability to stay focused and plan tasks effectively (directly and indirectly) predicts poor sleep outcomes. This relationship is mediated by excessive smartphone use, academic procrastination, and bedtime procrastination. Our findings are relevant in light of self-regulatory learning, which is crucial in medical education. This is a recursive cycle of planning, emotion regulation, proper strategy selection and self-monitoring. Future interventions addressing attentional and non-planning impulsivity, problematic smartphone use, academic procrastination, and in turn, bedtime procrastination might make this routine more effective. In the conclusion section, practical implications of the results are discussed.

## Background

The negative impact of sleep deprivation is well-known and widely studied. Insufficient sleep can lead to degraded cognitive functions, such as reduced attention and psychomotor vigilance [1, 2], negatively impacting emotion generation and regulation [3], and may be associated with lower quality of life and mental health symptoms such as depression and anxiety [4].

Poor sleep outcomes, such as delayed sleep patterns, decreased sleep timing, attentional deficits, and circadian rest-activity pattern disturbance have a positive relationship with impulsivity [5, 6] Impulsivity is suggestive of a person’s predisposition toward rapid, unplanned reactions to external or internal stimuli while ignoring the negative consequences of these behaviours [7]. According to the most widely accepted notion, it is a 3-dimensional construct, composed of motor impulsivity, attentional impulsivity, and non-planning impulsivity [8]. While the first type means acting without considering consequences, the second refers to difficulty focusing on a task, and the third one is focused on the present without planning for the future.

In the case of insufficient sleep, impulsivity might correlate with another consequence of poor sleep, namely, decreased cognitive function. In a study after partial sleep deprivation, the participants made faster reactions but also had less accurate responses in a cognitive performance test, indicating that sleep loss could lead to more impulsive reactions and decreased cognitive function. The subjects also reported reduced subjective performance after the deprivation period [9].

One possible mediator between impulsivity and poor sleep outcomes is bedtime procrastination, a situation when people fail to go to sleep at the intended time, with nothing hindering them from doing so [10]. First, a strong correlation between general procrastination and impulsivity has been demonstrated at the genetic level, supporting the proposal that procrastination may be an evolutionary by-product of impulsivity [11]. In line with this, impulsivity also had a positive relationship with bedtime procrastination, both as a standalone variable [12] and as part of a composite variable [10]. Second, several findings proved a positive association between bedtime procrastination and poor sleep outcomes such as daytime fatigue and sleep insufficiency [10, 13–16]. Furthermore, the possible link between poor cognitive performance and bedtime procrastination has not yet been studied, however, several findings suggest that insufficient sleep may affect cognitive and academic performance [17–19]. In line with this, bedtime smartphone use, a possible manifestation of bedtime procrastination, had a negative association with academic performance [20].

Many factors might mediate the relationship between impulsivity and bedtime procrastination; however, screen time could be one of the most prevalent factors in the digital age. This is particularly true in the case of university students for whom smartphone usage is essential in both their private and academic lives. As a result, people often go to bed later than they previously planned because they fail to resist the temptations originating from different media. In line with this assumption, lower self-regulation was found to be associated with bedtime procrastination, and this was partially mediated by television viewing [21]. Furthermore, individuals with a higher level of bedtime procrastination spent more time engaging in social and leisure activities using different forms of media three hours before bedtime [22]. The smartphone is a medium that might interfere with sleep, and its usage can often turn into behavioural addiction. Smartphone addiction is characterized by compulsive behavioural patterns, functional impairments, withdrawal symptoms when smartphone use is suspended, and tolerance manifesting in excessive use [23]. Excessive smartphone use is positively correlated with impulsivity [24], poor sleep quality [25], and bedtime procrastination [26–29]. Therefore, we suggest that smartphone usage could mediate the relationship between impulsivity and bedtime procrastination.

In higher education, more specific mediators should also be considered. In addition to the previously mentioned mediator, that is, problematic smartphone use, we believe that a specific type of procrastination, namely, academic procrastination, may mediate the relationship between impulsivity and bedtime procrastination among college students. Academic procrastination means mainly academic task delays, such as writing term papers, reading weekly assignments, or studying for exams [30]. Impulsivity was positively correlated with academic procrastination in previous studies [31, 32]. In line with this, self-monitoring and inhibition, two executive functions that hinder acting on impulse and maintaining self-regulation, were significant correlates of academic procrastination [33]. Moreover, it was also shown to have a direct effect on bedtime procrastination in a path analysis study [34]. Since smartphone addiction was positively related to academic procrastination in another study [35], we supposed that academic procrastination can also mediate the association between smartphone addiction and bedtime procrastination. Immersive activities such as social media use or studying near bedtime may lead to a lack of nighttime routines and in turn contribute to bedtime procrastination [36].

### The present study

**Fig. 1:**
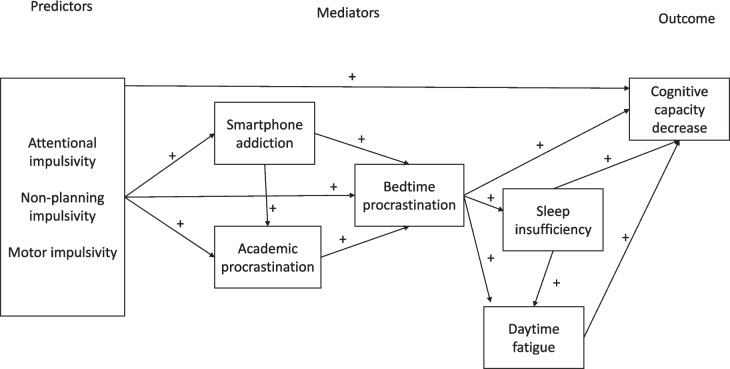
The hypothesized path model to explain the link between impulsivity and students’ bedtime procrastination

The major aim of this study is to integrate previous findings into one model. To the best of our knowledge, the proposed comprehensive conceptual model (see Fig. 1) is the first to include impulsivity, academic procrastination, addictive smartphone use, and bedtime procrastination together to determine their roles in insufficient sleep. In this study, we examined a sample of university students, more precisely, a special population of medical students, for several reasons. First, healthcare students, as a special subgroup of the general population, are particularly at risk for sleep disturbances due to their intensive and challenging training. In general, medical students around the world frequently complain about insomnia or sleepiness, and in a number of studies, the prevalence of sleeping problems was higher among medical students than in the general population [37]. The prevalence of bedtime procrastination was also higher for college students than for non-students and had a negative correlation with age [38]. These findings suggest that students at the beginning of their studies were especially at risk for sleep delay. In addition, self-regulation skills are crucial during academic years in medical school. Effective learning is based on cycles of self-regulatory processes involving planning and setting goals, regulating emotions and thoughts during the learning process, selecting proper learning strategies, and self-reflection by which students evaluate whether they achieved their previously set goals [39, 40]. Finally, younger people, especially college undergraduates, are more prone to problematic smartphone use in comparison to their older peers [41–43]. Unfortunately, despite its relevance, bedtime procrastination and its possible correlates have not yet been explored except for a study on the role of bedtime procrastination in medical students’ depressive symptoms [44]. In this regard, the current study should provide a unique contribution to this topic since managing bedtime and its correlates might be especially challenging for medical students.

Based on previous findings, we developed five hypotheses. Our first hypothesis supposes a direct relationship between the three impulsivity subtraits and bedtime procrastination. Our second hypothesis presumes an indirect relationship between the impulsivity variables and bedtime procrastination mediated by smartphone addiction and academic procrastination, respectively. Our third hypothesis proposes smartphone addiction as a predictor of academic procrastination; thus, we predict an indirect relationship between the impulsivity variables and bedtime procrastination with multiple serial mediations of smartphone addiction and academic procrastination. Our fourth hypothesis states that bedtime procrastination is a significant predictor of the outcome variables, including the subjective feeling of too little sleep, fatigue, and a decrease in cognitive capacity. Based on the previous hypotheses, our fifth hypothesis originally presumes an indirect relationship between the impulsivity variables and the sleep outcomes via the serial mediation of smartphone addiction, academic procrastination and bedtime procrastination. We note here that in our final model cognitive capacity decrease was the only outcome variable. Sleep insufficiency and daytime fatigue were considered additional mediators.

## Methods

### Participants and procedure

In accordance with the research aims, a convenience sample of medical students from several countries (*N* = 211; age_M_ = 22.15; age_SD_ = 3.47; 71.6% women) was selected at the Faculty of Medicine (English programme), University of Szeged, Hungary. Regarding the study population, based on the data from the relevant university body, more than half of the students in the 2021–2022 academic year were from Iran (20%), South Korea (13%), Israel (11%), Japan (10%), India (5%), and Jordan (4%) and the others from several more countries from the European, African or Asian regions, altogether 808 students. Thus, more than 25% of them participated in the study. Besides age and gender, however, we did not collect information on ethnicity or other sociodemographic and socioeconomic data due to being irrelevant to the topic. Respondents were from various academic years: 22.7 percent of them were first-year students, 25.1 percent were from second-year, 17.1 percent from third-year, 17.1 percent from fourth-year, 14.2 percent from fifth-year, and 3.8 percent from sixth-year. Since we decided to use the English-validated scales, all participants took part in the English programme attending courses in English. Data were collected via an online survey using Google Forms™ during the fall semester of the 2021–22 academic year. The hyperlink of the survey was shared on social media groups and online student platforms as well as additional online educational interfaces. The study was carried out in accordance with the Declaration of Helsinki and with the approval of the United Ethical Review Committee for Research in Psychology (EPKEB, Hungary) under #2021–89.

### Measures

#### Sociodemographic variables

We asked respondents’ gender (male/female/other) and age.

#### Impulsivity

The Abbreviated Impulsiveness Scale (ABIS) [45] was used to measure impulsiveness. The scale consists of three subscales: attentional impulsiveness with 3 reverse and 2 standard-scoring items (e.g., ‘I don’t “pay attention.”’), motor impulsiveness with four items (e.g., ‘I say things without thinking.’), and non-planning impulsiveness with four reverse-scoring items (e.g., ‘I am future-oriented.’). Items are answered on four-point Likert scales from 1 (rarely/never) to 4 (almost always/always).

For each subscale, the average of the scores for each item on the given scale is calculated after reverse-scoring the specified items. Cronbach’s alphas were 0.72 for non-planning impulsivity, 0.73 for motor impulsivity, and 0.71 for attentional impulsivity.

#### Bedtime procrastination

The Bedtime Procrastination Scale (BPS) [10] is a 9-item questionnaire used for assessing bedtime procrastination. The items (e.g., ‘I go to bed later than I had intended.’) can be answered on a five-point Likert scale from 1 ((almost) never) to 5 ((almost) always). After reverse-scoring items 2, 3, 7 and 9, the total BPS score is the average of the scores of the individual items. The Cronbach’s alpha was 0.87 for our data.

#### Smartphone addiction

The Smartphone Addiction Scale Short Version (SAS-SV) [46] is a 10-item scale (e.g., ‘Missing planned work due to smartphone use.’) that measures smartphone addiction with a six-point Likert scale from 1 (strongly disagree) to 6 (strongly agree). Cronbach’s alpha was found to be 0.83 in our data.

#### Academic procrastination

We measured students’ procrastination on academic tasks by the Academic Procrastination Scale-Short Form (APS-SF) [47]. The instrument contains five items (e.g., ‘I know I should work on schoolwork, but I just don’t do it.’) that can be assessed with a 5-point Likert scale from 1 (disagree) to 5 (agree). Cronbach’s alpha was.90 on our data.

#### Sleep outcome variables

Regarding daytime fatigue, we asked the following: ‘On average, how many days in a typical week of the education period do you feel tired?’ In relation to sleep insufficiency, we asked the following: ‘On average, how many days in a typical week of the education period do you feel you have slept too little?’ These questions are based on the study of Kroese, De Ridder, Evers, and Adriaanse [10]. In our study, however, respondents could mark a concrete number by using a scroll bar. We also examined the participants’ subjective cognitive capacity decrease by asking the following: ‘On average, how many days in a typical week of the education period do you feel your cognitive capacity is unsatisfactory, i.e., you cannot pay attention to your studies, memorize the material effectively, interpret the material, etc.?’.

### Data analysis

The dimensionality of the scales was investigated by confirmatory factor analysis (CFA) by applying the diagonally weighted least squares estimation method. In the case of the BPS, SAS-SV and APS-SF, a single-component model with one latent variable was tested, whereas in the case of the ABIS, a three-component model was examined with attentional, motor and non-planning latent factors. The model fit criteria were a nonsignificant chi-square value, root mean square error of approximation (RMSEA) ≤ 0.06, comparative fit index (CFI) ≥ 0.95, and standardized root mean square residual (SRMR) ≤ 0.08 [48]. CFAs were conducted by ROP-R, a user-friendly statistical software [49] that runs scripts of the R package [50].

IBM SPSS Statistics 25.0 for Windows [51] was used to conduct a correlation analysis of the study variables. Since the respondents had to answer all the questions, our data did not contain any missing cases. The primary analytical strategy for our research was a path analysis with a maximum likelihood estimation method to test the hypothesized model using IBM SPSS AMOS 24.0. for Windows [52]. Nonsignificant chi-square value, root mean square error of approximation (RMSEA) ≤ 0.06, comparative fit index (CFI) ≥ 0.95, and standardized root mean square residual (SRMR) ≤ 0.08 were used to determine acceptable model fit [48]. Modification indices were also taken into consideration in the development of the final model. Coefficients of direct and indirect effects were estimated with a bias-corrected percentile method, 2000 bootstrap samples, and a 95% confidence interval. The hypothesized special indirect effects were tested with the User-defined estimands function of IBM SPSS AMOS 24.0 for Windows [52] and they were considered significant if the confidence interval did not pass zero. First, we investigated the possible indirect effects of impulsivity traits on bedtime procrastination with serial mediation of smartphone addiction and academic procrastination, testing our first, second and third hypotheses. Second, to test our fourth and fifth hypotheses, we examined the indirect effects of impulsivity traits on cognitive capacity decrease, which was the outcome variable in the final model. We hypothesized the serial mediation of smartphone addiction, academic procrastination, bedtime procrastination, sleep insufficiency and daytime fatigue.

## Results

### Confirmatory factor analysis of the scales

The three-component structure of ABIS was verified on our dataset. Fit indices showed a good model fit with χ^2^ = 64.49, df = 62, *p* > 0.05, RMSEA = 0.01, CFI = 0.99, SRMR = 0.06. Standardized factor loadings ranged from 0.31 to 0.67, 0.51 to 0.76, and 0.53 to 0.82 for attentional impulsivity, motor impulsivity and non-planning impulsivity, respectively.

The unifactorial model of BPS had good model fit indices on our data with χ^2^ = 26.58, df = 27, *p* > 0.05, RMSEA = 0.00, CFI = 1, SRMR = 0.05, verifying the original model with one latent factor. Standardized factor loadings ranged from 0.48 to 0.79.

CFA of SAS-SV also indicated an overall good model fit with χ^2^ = 49.90, df = 35, *p* < 0.05, RMSEA = 0.05, CFI = 0.98, SRMR = 0.06, confirming the original one-component structure of SAS-SV on our data. We note here that* p* was 0.049, close to the 0.05 probability level. Standardized factor loadings ranged from 0.52 to 0.65.

Finally, the one-component model of the APS-SF was also verified on our data with χ^2^ = 8.66, df = 5, *p* > 0.05, RMSEA = 0.059, CFI = 0.996, SRMR = 0.05, indicating good model fit. Standardized factor loadings ranged from 0.70 to 0.85.

### Correlation of the examined variables

Table 1 displays the correlation matrix for the examined variables. Regarding our hypotheses, the most important result is that bedtime procrastination was significantly correlated with all variables, except for gender and age. It showed the strongest correlation with academic procrastination (r = 0.41, *p* < 0.001). Among the impulsivity dimensions, attentional impulsivity displayed a correlation with all variables, except age and gender; motor impulsivity was correlated with smartphone addiction (r = 0.14, *p* < 0.001) and academic procrastination (r = 0.30, *p* < 0.001), while non-planning impulsivity had a significant relationship with academic procrastination (r = 0.45, *p* < 0.001).

**Table 1:** Bivariate correlations of the measured variables

	1	2	3	4	5	6	7	8	9	10	11
1. Bedtime procrastination	1										
2. Attentional impulsivity	.29^***^	1									
3. Motor impulsivity	.14^*^	.39^***^	1								
4. Non-planning impulsivity	.25^***^	.58^***^	.40^***^	1							
5. Smartphone addiction	.28^***^	.30^***^	.14^*^	.11	1						
6. Academic procrastination	.41^***^	.52^***^	.30^***^	.45^***^	.39^***^	1					
7. Fatigue (days a week)	.27^***^	.19^**^	.11	.04	.12	.11	1				
8. Sleep insufficiency (days a week)	.36^***^	.22^**^	.11	.07	.16^*^	.21^**^	.64^***^	1			
9. Cognitive capacity decrease (days a week)	.22^***^	.33^***^	.10	.04	.14^*^	.29^***^	.49^***^	.50^***^	1		
10. Gender	-.01	-.02	-.10	-.03	.01	.01	.12	-.01	.03	1	
11. Age	-.12	-.10	-.08	-.07	-.16^*^	-.07	.08	.01	.01	-.14	1

^*^p < .05, **p < .01, ***p < .001

### Path analysis

Our preliminary path analysis resulted in a significant chi-square goodness to fit with χ^2^ = 199.15, df = 19, *p* < 0.001; furthermore, RMSEA = 0.21; CFI = 0.65; SRMR = 0.13, indicating poor model fit. Taking into consideration the modification indices, we added the following plausible paths to the initial model: a path from attentional impulsivity to cognitive capacity decrease; from non-planning impulsivity to cognitive capacity decrease; from fatigue to cognitive capacity decrease; from sleep insufficiency to cognitive capacity decrease; and from sleep insufficiency to daytime fatigue. While controlling for basic demographics (gender, age), a path analysis of the expanded model had a non-significant chi-square with χ^2^ = 21.64, df = 13, *p* = 0.06 demonstrating a good model fit. Other fit measures also suggested a well-fitted model with RMSEA = 0.056; CFI = 0.98 and SRMR = 0.03. Table 2 presents the unstandardized estimates of the paths within this model.

**Table 2:** Unstandardized parameters of path analysis

Path	Estimate	SE	CR	P
Motor impulsiveness → Academic procrastination	0.61	0.54	1.13	.26
Attentional impulsiveness → Academic procrastination	2.87	0.73	3.95	.00
Non-planning impulsiveness → Academic procrastination	1.93	0.60	3.23	.00
Attentional impulsiveness → Smartphone addiction	4.59	1.14	4.03	.00
Motor impulsiveness → Smartphone addiction	0.43	0.88	0.50	.62
Smartphone addiction → Academic procrastination	0.20	0.04	4.67	.00
Non-planning impulsiveness → Smartphone addiction	-1.24	0.964	-1.29	.20
Smartphone addiction → Bedtime procrastination	0.02	0.01	1.87	.06
Academic procrastination → Bedtime procrastination	0.05	0.01	3.88	.00
Non-planning impulsiveness → Bedtime procrastination	0.10	0.11	0.88	.38
Attentional impulsiveness → Bedtime procrastination	0.09	0.14	0.65	.52
Motor impulsiveness → Bedtime procrastination	-0.04	0.10	-0.35	.72
Bedtime procrastination → Sleep insufficiency	0.78	0.14	5.63	.00
Bedtime procrastination → Fatigue	0.11	0.11	0.97	.33
Sleep insufficiency → Fatigue	0.59	0.05	11.14	.00
Bedtime procrastination → Cognitive capacity decrease	0.03	0.13	0.21	.84
Attentional impulsiveness → Cognitive capacity decrease	1.09	0.24	4.63	.00
Sleep insufficiency → Cognitive capactity decrease	0.26	0.07	3.59	.00
Fatigue → Cognitive capacity decrease	0.27	0.08	3,55	.00
Non-planning impulsiveness → Cognitive capacity decrease	-0.52	0.20	-2.64	.01

Abbreviations: *SE* Standard error of regression weight estimate, *CR* Critical Ratio

Attentional impulsivity was a predictor of cognitive capacity decrease (β = 0.33,* p* < 0.001), whereas non-planning impulsivity was a negative predictor of it (β = -0.19, *p* < 0.01).

Regarding our first hypothesis, we proposed a direct path between each type of impulsivity and bedtime procrastination. Nevertheless, neither motor (β = -0,25, *p* = 0.72), nor non-planning (β = 0.07, *p* = 0.38), nor attentional impulsiveness (β = 0.05, *p* = 0.52) proved to be significant predictors of bedtime procrastination.

According to our second hypothesis, we expected an indirect path between each impulsivity dimension and bedtime procrastination mediated by smartphone addiction and academic procrastination. The paths from motor impulsivity to smartphone addiction (β = 0.04, *p* = 0.62) and academic procrastination (β = 0.7, *p* = *0.2*6) were not significant, thus motor impulsiveness was excluded from the analysis of indirect effects. Attentional impulsivity was associated with smartphone addiction (β = 0.33, *p* < 0.001) and academic procrastination (β = 0.28, *p* < 0.001). Non-planning impulsivity was a significant predictor of academic procrastination (β = 0.22, *p* < 0.001), while academic procrastination was a significant predictor of bedtime procrastination (β = 0.30, *p* < 0.001).

The significant special indirect effects (see Table 3) partly supported our second hypothesis. An indirect effect of attentional impulsivity on bedtime procrastination mediated by academic procrastination was found (estimate = 0.14, *p* < 0.001). The indirect effect of non-planning impulsivity on bedtime procrastination with the mediation of academic procrastination also proved to be significant (estimate = 0.09, *p* < 0.001).

**Table 3:** Estimates of special indirect effects between impulsivity factors and bedtime procrastination

Indirect effect	Estimate	Lower	Upper	P
Attentional impulsivity → Academic procrastination → Bedtime procrastination	0.14	0.05	0.29	.00
Non-planning impulsivity → Academic procrastination → Bedtime procrastination	0.09	0.03	0.20	.00
Attentional impulsivity → Smartphone addiction → Academic procrastination → Bedtime procrastination	0.04	0.02	0.11	.00

The third hypothesis suggests a path from smartphone addiction to academic procrastination and, consequently, multiple indirect effects from the impulsivity dimensions to bedtime procrastination, mediated by smartphone addiction and academic procrastination. First, smartphone addiction was indeed a significant predictor of academic procrastination (β = 0.27, *p* < 0.001). Furthermore, the hypothesized multiple indirect effects (see Table 3) from attentional impulsivity to bedtime procrastination via smartphone addiction and academic procrastination were significant (estimate = 0.04, *p* < 0.001).

A significant path from bedtime procrastination to sleep insufficiency (β = 0.37, *p* < 0.001) partly confirmed our fourth hypothesis. Significant paths were also found from sleep insufficiency to daytime fatigue (β = 0.62, *p* < 0.001) and cognitive capacity decrease (β = 0.27, *p* < 0.001), and finally, from daytime fatigue to cognitive capacity decrease (β = 0.27, *p* < 0.001).

Table 4 displays special indirect effects from attentional and non-planning impulsivity to the final outcome, cognitive capacity decrease with the serial mediation of smartphone addiction, academic procrastination, bedtime procrastination, sleep insufficiency and fatigue. All of these possible indirect effects were statistically significant at the *p* < 0.01 level, supporting our fifth hypothesis.

**Table 4:** Estimates of special indirect effects between impulsivity factors and cognitive capacity decrease as the final outcome

Indirect effect	Estimate	Lower	Upper	P
Attentional impulsivity → Smartphone addiction → Academic procrastination → Bedtime procrastination Sleep insufficiency → Fatigue → Cognitive capacity decrease	0.005	0.002	0.02	.001
Attentional impulsivity → Smartphone addiction → Academic procrastination → Bedtime procrastination → Sleep insufficiency → Cognitive capacity decrease	0.009	0.003	0.024	.001
Attentional impulsivity → Bedtime procrastination → Sleep insufficiency → Fatigue → Cognitive capacity decrease	0.017	0.006	0.045	.001
Attentional impulsivity → Academic procrastination → Bedtime procrastination → Sleep insufficiency → Cognitive capacity decrease	0.028	0.010	0.069	.001
Non-planning impulsivity → Academic procrastination → Bedtime procrastination → Sleep insufficiency → Fatigue → Cognitive capacity decrease	0.011	0.004	0.030	.001
Non-planning impulsivity → Academic procrastination → Bedtime procrastination → Sleep insufficiency → Cognitive capacity decrease	0.019	0.007	0.047	.001

**Fig. 2:**
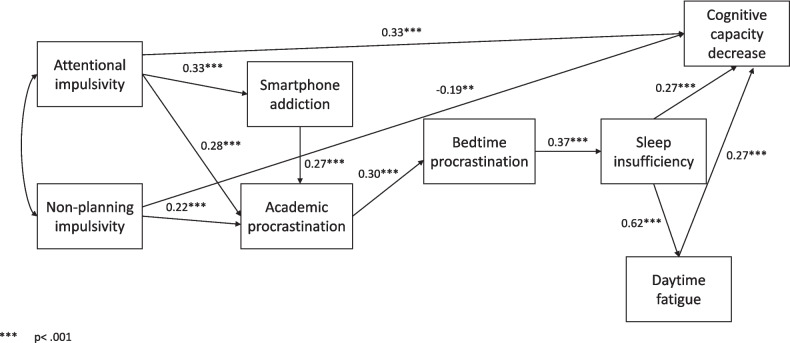
The final model of bedtime procrastination with significant paths and their standardized estimates

Figure 2 displays the final model with the standardized estimates of the significant paths.

## Discussion

The goal of our study was to develop a model of possible predictors of bedtime procrastination as well as outcomes of insufficient sleep. There is a well-known link between impulsivity and different aspects of sleep disturbance [5, 6]; thus attentional, motor and non-planning impulsivity were the predictors of our model. We also expected that academic procrastination, excessive smartphone use and bedtime procrastination might be mediators between impulsivity and sleep outcomes. Bedtime procrastination, like other forms of procrastination, is considered a manifestation of failed self-regulation [10, 53–55]. Previously, impulsivity was found to be an important correlate of low self-regulation, and in turn, it was strongly associated with procrastination [56, 57] and bedtime procrastination [12]. Based on previous findings [31, 32, 34], we also included academic procrastination and smartphone addiction as possible mediators between impulsivity and bedtime procrastination. Furthermore, we involved a sample of medical students, since their studies require high self-regulation and impulse control, and the intensive and demanding university years put them at particular risk of sleep disturbances [37].

We found a direct relationship between attentional impulsivity and the final outcome, cognitive capacity decrease, and a negative direct link between non-planning impulsivity and cognitive capacity decrease. This later negative relationship at first glance contradicts the general consensus and previous findings [58–60] that suggest a positive association between different types of impulsivity and problematic cognitive functioning. However, non-planning impulsivity refers to paying attention to the present studies, that is, current cognitive processes, instead of planning for the future.

Contrary to our prior presumptions, we did not find a direct path between the subtraits of impulsivity and bedtime procrastination. Nevertheless, both attentional and non-planning impulsivity had an indirect effect on bedtime procrastination mediated by academic procrastination. These findings replicate and integrate into a single model previous results on the relationship between different aspects of self-regulation and academic procrastination [61, 62], as well as the relationship between academic procrastination and bedtime procrastination [34]. Our finding that attentional impulsivity is indirectly associated with bedtime procrastination is in line with other study results showing a positive relationship between attentional deficits and procrastination [63], bedtime procrastination [64], and between inattention and bedtime procrastination [65]. In our study, the other indirect path between non-planning impulsivity and bedtime procrastination fits the assumptions that highlight the importance of planning and goal setting in overcoming procrastination during the school years. For example, the implementation intention supports goal attainment by breaking down academic tasks into simple “if–then” formats that describe the concrete activities needed under the given circumstances [66]. A similar goal clarification intervention based on the SMART method successfully reduced college students’ impulsivity and procrastination. The SMART method stands for setting specific, measurable, agreed upon, realistic and time-based goals [67]. Likewise, online implementation intention exercises successfully reduced bedtime procrastination compared to the control group who applied positive thinking techniques [68].

Moreover, we found multiple serial indirect effects from attentional impulsivity via smartphone addiction and academic procrastination to bedtime procrastination, which is in line with previous findings that show a positive relationship between smartphone addiction and students’ procrastination [35, 69–71], as well as between academic procrastination and bedtime procrastination [34]. Attentional impulsivity was a significant predictor of smartphone addiction and academic procrastination, which is consistent with a prior study where students’ distraction cognition was positively associated with procrastination and problematic smartphone use [69].

In our model, bedtime procrastination predicts students’ subjective complaints about too little sleep, which in turn is a predictor of fatigue and cognitive capacity decrease. This result contributes to the growing body of evidence linking bedtime procrastination to insufficient sleep quality and duration [10, 38, 72]. It also draws attention to the evidence of the negative effects of sleep deprivation on cognitive function [1, 2, 73]. Several studies have reported similar findings on medical students, indicating that sleeping problems and poor sleep quality adversely affect their academic performance [74–77].

Finally, these findings can be integrated into a final model, where attentional and non-planning impulsivity have not only a direct relationship with the final sleep outcome, that is, cognitive capacity decrease but also indirect ones. These multiple indirect effects are mediated by smartphone addiction, academic procrastination, bedtime procrastination, sleep insufficiency and fatigue.

Our study is not without limitations. First, we note that the students’ academic year was not distributed equally across all cohorts, and due to this bias, sixth-year students were underrepresented. We assigned different academic years to our sample, although the academic load varies from year to year. Based on this limitation, a hypothesis to consider for future research is that students’ bedtime procrastination varies over the academic years due to the altering demands. A second limitation is the cross-sectional study design; therefore, cause-and-effect relationships cannot be justified between the variables. Among the limitations, we must also mention that only a modified version of our a priori model proved to be acceptable. Future research should test this modified model, including direct paths between impulsivity and sleep outcomes, and treat cognitive capacity decrease as the final outcome. The involved sample should be larger to make the results more reliable. Finally, impulsivity was measured using self-administered scales, although other types of measures could have been used as well. Future research may want to address the link between impulsivity, the mediators and the cognitive capacity decrease by applying more objective measures of impulsivity, i.e., the go/no-go task paradigm, similar to studies focusing on general procrastination (e.g., [63]).

## Conclusions

Medical schools put an enormous academic load on students due to the attainment of medical knowledge and skills that need self-regulated learning, cycles of interpretation of the demands, planning, selecting adaptive strategies, adjusting them based on self-monitoring, and evaluating how successful the learning is [40]. During this tense daily routine, overcoming impulses and finding and keeping the right time for going to bed can be critical. Our results provide insight into this challenge, showing that students’ attentional impulsivity has an indirect relationship with bedtime procrastination via the serial mediation of problematic smartphone use and academic procrastination. We also found an indirect association between non-planning impulsivity and bedtime procrastination via academic procrastination.

This study also provides a perspective on the prevention and intervention of sleep disturbances, chronic fatigue and cognitive capacity decrease in medical schools. Our findings contribute to future interventions addressing medical students’ daily management and bedtime procrastination. First, we have to explore sleep patterns during the academic years and the factors affecting them. In addition, we should focus on the development of self-regulatory skills that improve planning and setting academic goals, e.g., introducing self-control training. Improving attention and excluding distracting stimuli (e.g., smartphones) should also be part of such an intervention, as these efforts can reduce both academic and bedtime procrastination. Finally, this intervention should adapt modules from other evidence-based trainings that successfully targeted the mediators of our model, i.e., smartphone addiction, academic procrastination and bedtime procrastination. In the following paragraphs, we outline some of them.

Based on a meta-analysis, intervention programmes on smartphone addiction can be classified into 10 types, namely, psychological, social support, lifestyle, technological, family, medical, educational, exercise, mindfulness, and meditation interventions, of which psychological interventions are the most commonly applied methods [78]. At the same time, the number of e-health intervention programmes is growing as well, made up of goal setting, personalized feedback, mindfulness, and behavioural suggestions sent by a cellphone application [79].

Since procrastinators usually have irrational beliefs and negative thoughts, academic procrastination can be successfully managed by cognitive behavioural methods [80]. For example, cognitive behavioural modules of the Clinical Centre of Interventions help clientsunderstand their unhelpful rules/assumptions, discomfort driven by the task, procrastination excuses and procrastination activities that hinder them from achieving their goals. The programme also offers practical techniques to stop procrastination, e.g., replacing unhelpful subjective rules with more adaptive ones [81].

Recently, there have been some aspirations to develop bedtime procrastination-specific interventions. Mental contrasting is a self-regulation method in which people establish their goals (i.e., going to bed in time) pairing it with a pleasant outcome (i.e., waking up fresh). Then, they imagine a possible obstacle which can hinder their ability to reach this goal. Mental contrasting successfully increased commitment to reduce bedtime procrastination and decreased the average delay of going to bed [68]. Another intervention that successfully improved bedtime procrastination was based on the transtheoretical model, motivational interview techniques and behaviour modification principles. For example, participants learned to connect the desired behaviour (decreasing bedtime procrastination) with their values. During a functional analysis they described factors that preceded bedtime procrastination, their specific behaviours during the delay in going to bed and the reinforcements of this problematic behaviour [82].

## Data Availability

The datasets used and/or analysed during the current study are available from the corresponding author on reasonable request.
